# Novel Dual-Layer Zwitterionic Modification of Electrospun Nanofibrous Membrane for Produced Water Treatment and Reclamation

**DOI:** 10.3390/membranes15080244

**Published:** 2025-08-10

**Authors:** Sunith B. Madduri, Raghava R. Kommalapati

**Affiliations:** 1 Center for Energy and Environmental Sustainability, Prairie View A&M University, Prairie View, TX 77446, USA; sbmadduri@pvamu.edu; 2 Department of Civil and Environmental Engineering, Prairie View A&M University, Prairie View, TX 77446, USA

**Keywords:** electrospinning, nanofibrous dual-layer membrane, zwitterionic surface modification, produced water treatment, sustainable water reuse

## Abstract

Produced water, a byproduct of oil and gas extraction, poses significant environmental challenges due to its complex composition and high salinity. Conventional treatment technologies often struggle to achieve efficient contaminant removal while maintaining long-term operational stability. Membrane-based separation processes, particularly forward osmosis (FO), offer a promising alternative due to their low hydraulic pressure requirements, high selectivity, and ability to mitigate fouling and scaling effects. This study fabricated and evaluated a novel dual-layer zwitterion-modified electrospun nanofibrous membrane for enhanced produced water (PW) treatment. The dual-layer design consists of a highly porous electrospun nanofibrous support layer for improved permeability and mechanical strength, coupled with a zwitterionic-modified selective layer to enhance antifouling properties and selective contaminant rejection. The zwitterionic surface modification imparts superior hydration capacity, reducing organic and biological fouling while improving water transport efficiency. The membranes are characterized using scanning electron microscopy (SEM), thermogravimetric analysis (TGA), Fourier Transform Infrared (FTIR) spectroscopy, X-ray diffraction (XRD), contact angle and tensile strength measurements, and nuclear magnetic resonance (NMR) spectroscopy to assess their morphological, structural, and chemical properties. The performance evaluations demonstrated significantly higher water flux (up to 16.05 L m^−2^ h^−1^ for SPW (synthetic produced water) and 6.00 L m^−2^ h^−1^ for PW using NaBr) and excellent solid rejection (up to 96.02% for SPW and 88.90% for PW), reduced concentration polarization, and superior antifouling performance compared to conventional FO membranes. Experimental results from bench-scale trials demonstrate that this advanced membrane technology offers enhanced water recovery and contaminant removal efficiency, making it a viable solution for industrial-scale PW treatment and reuse. The findings underscore the potential of next-generation dual-layer FO membranes in promoting sustainable water resource management within the oil and gas sector while minimizing environmental impact.

## 1. Introduction

Produced water is the most significant volume waste stream associated with oil and gas extraction, containing a complex mixture of organic and inorganic contaminants that pose significant environmental and health risks. This wastewater, generated from conventional and unconventional extraction processes, contains hydrocarbons, heavy metals, salts, and chemical additives used in drilling operations [1,2]. As global oil and gas activities continue to expand, the management of produced water (PW) presents a growing challenge, necessitating the development and implementation of efficient and sustainable treatment technologies. The environmental impact of untreated PW is severe, as it can lead to pollution of surface and groundwater resources, disrupt aquatic ecosystems, and pose risks to human health through contamination of food and water supplies [3,4]. The treatment of PW is complicated by various contaminants, including dissolved organic compounds and trace elements, which are difficult to remove using conventional treatment methods such as chemical precipitation, coagulation, and filtration [5,6]. Furthermore, the high salinity of PW presents additional challenges for treatment and limits its potential for reuse [4]. Consequently, there is a critical need for innovative water treatment technologies that can efficiently address these challenges.

Electrospun nanofibrous membranes have attracted increasing attention recently due to their high porosity, large surface area-to-volume ratio, and tunable pore size distribution [7,8]. These properties make them promising candidates for various water treatment applications, including the treatment and reuse of PW [9]. Moreover, the functionalization of electrospun membranes with zwitterionic materials offers additional advantages, such as enhanced fouling resistance and selective adsorption capabilities [10,11]. Fabricating electrospun nanofibrous membranes involves electrospinning, which utilizes an electric field to draw polymer solutions or melts into fine fibers [12,13]. Typically, polymers such as polyvinylidene fluoride (PVDF), polyacrylonitrile (PAN), and polyethylene oxide (PEO) are dissolved in suitable solvents to form polymer solutions [14,15]. The electrospinning setup consists of a high-voltage power supply, a syringe pump for controlled solution delivery, and a grounded collector. Under high voltage, the polymer solution is ejected from a syringe tip connected to the positive electrode [7,13]. Electrostatic forces overcome the surface tension of the solution, forming a Taylor cone and initiating the stretching and solidification of the polymer jet. Continuous nanofibers are deposited onto the grounded collector as the solvent evaporates, forming a nonwoven membrane structure [8,12].

The zwitterionic modification involves functionalizing electrospun nanofibrous membranes with zwitterionic polymers or molecules that contain positive and negative functional groups within the same molecule [16,17]. Zwitterionic materials exhibit unique properties, including intense hydration, antifouling behavior, and low protein adsorption, making them ideal candidates for membrane modification [17]. The modification typically involves surface grafting or blending zwitterionic polymers onto electrospun membranes. Common zwitterionic molecules used for modification include sulfobetaine, carboxybetaine, and phosphorylcholine [18,19]. This modification enhances electrospun membranes’ hydrophilicity and antifouling properties, reducing fouling and improving water permeability. The performance of electrospun nanofibrous membranes and zwitterionic modifications for PW treatment is evaluated based on various parameters, including water flux, rejection efficiency, fouling resistance, and long-term stability [10,18,19]. Bench-scale and pilot-scale experiments assess the feasibility and scalability of the membrane technology. Electrospun nanofibrous membranes demonstrate promising results in removing hydrocarbons, heavy metals, and suspended solids from PW while maintaining high water flux and minimal fouling [11,20]. Additionally, zwitterionic modifications enhance the membrane’s antifouling properties and selectivity for specific contaminants, making them suitable for challenging water treatment applications [17]. As research advances, electrospun membranes with zwitterionic functionalization hold great potential for sustainable and efficient PW treatment and reuse [21,22]. Interfacial polymerization (IP) is a commonly employed method in the production of membranes to create thin, selective layers exhibiting high perm selectivity. It entails a swift polycondensation reaction occurring at the boundary of two non-mixing phases, usually an aqueous solution with a diamine monomer and an organic solvent with a tri-functional acyl chloride. This technique enables the production of ultrathin polyamide coatings with regulated thickness and crosslinking density, making it suitable for uses like forward osmosis and reverse osmosis. In the present research, IP was utilized to create the active selective layer on the electrospun support, improving both rejection performance and mechanical strength of the dual-layer membrane [23,24].

To further optimize the performance of electrospun membranes for PW treatment, researchers are exploring novel polymer compositions [25,26], fiber alignment techniques [27,28], and hybrid membrane structures [7,11]. The incorporation of advanced nanomaterials, such as metal–organic frameworks (MOFs), carbon nanotubes (CNTs), and functionalized graphene oxide, has shown promise in enhancing adsorption capacity, mechanical strength, and chemical resistance [29]. Moreover, computational modeling and machine learning approaches are employed to predict membrane performance and optimize fabrication parameters, reducing the need for extensive trial-and-error experimentation [30,31]. Integrating electrospun membranes with hybrid treatment processes, such as forward osmosis (FO) and membrane distillation (MD), is also being investigated to improve water recovery rates and minimize energy consumption. FO is the flow of water, through osmotic pressure, through a membrane from a low-saline liquid (feed solute) to a high-saline fluid (draw solute) [32]. The process of forward osmosis is ideal because it is a natural process, meaning the application of this process for wastewater treatment would require little to no energy compared to reverse osmosis, which would generally require pressure placed on the feed solute to force the liquid through the membrane [33]. The problem with using this process for the filtration of PW is the fact that the process of FO needs a membrane with high flux resistance and a carefully chosen draw solute, especially with the process aimed at treating PW, which can have a salinity much higher than that of even salt water [34]. Research needs to be focused on improvements to these FO drawbacks to create a process that can efficiently treat PW to meet the coming demand for fresh water.

As advancements in membrane science continue, electrospun nanofibrous membranes with zwitterionic functionalization are poised to play a pivotal role in addressing global water scarcity challenges through the efficient treatment and reuse of PW [19]. The current research presents a novel method for tackling the difficulties associated with PW treatment, an important byproduct of oil and gas extraction. Acknowledging the ongoing challenges of traditional membrane technologies, including their vulnerability to fouling, poor rejection efficiency, and reduced long-term performance, this study thoughtfully integrates cutting-edge electrospinning methods and dual-layer zwitterionic functionalization to significantly enhance the current membrane technology for sustainable water management. This research introduces a unique dual-layer design, combining an electrospun nanofibrous support layer featuring a highly porous structure with an advanced active zwitterionic-functionalized and selective active layer. The dual-layer configuration efficiently utilizes the advantages of both elements: the structural and mechanical strength provided by the nanofibrous substrate and the excellent antifouling features granted by the zwitterionic coatings. Significantly, this pairing is unparalleled in forward osmosis (FO) membrane studies, where zwitterionic coatings have generally been confined to single-layer membrane setups. Additionally, the created membranes contain accurately optimized levels of reduced graphene oxide (rGO) within the polymer matrix. Incorporating rGO greatly improves thermal stability, mechanical strength, and membrane permeability, whereas the carefully designed zwitterionic coatings provide exceptional hydrophilicity, decreased organic fouling, and extended operational stability. This synergistic combination of graphene-derived nanomaterials with zwitterionic chemistry clearly sets this study apart from earlier research, which typically concentrated on either incorporating nanomaterials or the functionalization with zwitterionic groups separately. Another essential innovation is the customized synthesis and integration of zwitterionic moieties, particularly sulfobetaine methacrylate (SBMA) and methacryloyloxyethyl phosphorylcholine thiol (MPC-SH), chemically bonded to polydopamine (PDA)-modified surfaces. This focused surface engineering greatly enhances the selective dismissal of impurities, an essential factor for large-scale reuse applications, and results in considerable advancements in water flux and solid rejection compared to traditional FO membranes. The performance of these membranes was systematically assessed in an FO system, where key operational parameters, including water flux, contaminant rejection efficiency, fouling resistance, and long-term stability, were analyzed. Additionally, bench-scale experiments are conducted to explore the scalability and feasibility of these membranes for industrial applications. This comprehensive and inclusive approach clearly sets the study to enhance sustainable water reuse practices significantly, offering important insights for industry experts and researchers seeking advanced membrane solutions in complex environmental and industrial settings.

## 2. Materials and Methods

### 2.1. Chemicals and Reagents

The materials used in this study were purchased from Sigma Aldrich (St. Louis, MO, USA), Alfa Aiser (Ward Hill, MA, USA), Fisher Scientific (Waltham, MA, USA), Fisher Chemical (Pittsburgh, PA, USA) and TCI America (Portland, OR, USA). The membrane fabrication process utilized Polyethyleneimine (PEI) Powder, N-methyl-2-pyrrolidone (NMP) solvent, N-dimethylformamide (DMF) solvent, Lithium chloride, and reduced graphene oxide (rGO), Active layer solvent 1: Heptane (99%) and trimesoyl chloride (TMC, 98%). Active layer solvent 2: M-phenylenediamine (MPD, 99%), trimesoyl chloride (TMC, 98%), camphorsulfonic acid (CSA), trimethylamine (TEA), and sodium dodecyl sulfate (SDS). Heptane (99%) and trimesoyl chloride (TMC, 98%) Zwitterionic coating 1: Dopamine hydrochloride, Trizma hydrochloride (Trizma-HCl), Sulfobetaine methacrylate (SBMA), and ethanol, Zwitterionic coating 2: Diisopropylamine (99.5%), 2-Methacryloyloxyethyl phosphorylcholine (MPC, 97%), dopamine hydrochloride (99%), Triethylamine (>99%), tris(hydroxymethyl)aminomethane (tris, >99.8%) 1, 10-decanedithiol (HS-C10-SH), and degassed chloroform, Synthetic produced water: Sodium chloride (NaCl), Sodium bicarbonate (NaHCO_3_), Sodium carbonate (Na_2_CO_3_), Sodium sulfate (Na_2_SO_4_), Calcium chloride (CaCl_2_), Magnesium chloride hexahydrate (MgCl_2·_6H_2_O), Sodium dodecyl sulfate (SDS), Permian basin produced water (TDS: 121.5 g/L) used in this study was diluted five times before use.

### 2.2. Membrane Fabrication by Electrospinning

The membrane fabrication process was started by preparing the polymer substrates for electrospinning. 58.2 wt.% of NMP was mixed with 25.2 wt.% of DMF solvent, and 16 wt.% of PEI polymer was dissolved by stirring at 80 °C. The solution was mixed evenly for 48 h until the powder was fully dissolved. A trace amount of Lithium chloride was added to the solution and allowed to mix. rGO loading of 0.25 wt.% was added to the polymer solution after optimization. The polymer solution was allowed to mix until it was homogeneously dispersed.

The electrospinning process involves four main steps: (i) charging the liquid and forming a Taylor cone, (ii) extending the charged jet in a straight line, (iii) thinning the jet as it whips due to an electric field and instability, (iv) solidification and collection of fibers on the grounded collector [35]. Electrospinning uses electrostatic forces to produce solid fibers from a liquid solution [36]. Forming a Taylor cone is a critical step during the electrospinning process [37]. When a liquid droplet hanging from a needle tip is introduced to a strong electric field, charge builds up on its surface [38]. The electrostatic repulsion generated by the surface charge works against the surface tension holding the droplet together. As more charge accumulates, the droplet becomes increasingly elongated until it reaches a critical point where the electrostatic forces overcome the surface tension [13,39]. At this stage, the droplet tip forms a conical protrusion known as a Taylor cone [39]. The cone shape represents an equilibrium between the concentrating electrostatic forces at the tip and the diminishing surface tension forces along the droplet. When the electrostatic forces fully overcome the surface tension, a liquid jet is ejected from the apex of the Taylor cone [37]. Solvent evaporation starts solidifying the jet while maintaining its linear trajectory. However, the straight configuration is volatile, representing a balance between outward electrostatic repulsion and inward surface tension. The electrostatic forces quickly overcome the surface tension, and the jet begins to bend and whip [40]. This brief extension in a straight line is critical for initiating the jet thinning and drawing, enabling uniform, continuous polymer fibers to form. The linear jet trajectory sets the foundation for the whipping instability needed to elongate and solidify the jet into fibers collected on the electrode [41]. The thinning of the jet during electrospinning refers to the process by which the charged liquid jet becomes progressively thinner in diameter as it travels from the spinneret to the collector [7,35,41]. As the charged jet travels through the electric field after ejecting from the Taylor Cone, it undergoes a significant reduction in diameter through a combination of elongation and solvent evaporation. The electrostatic repulsion causes the whipping jet to stretch and extend, reducing its cross-sectional area rapidly [41,42]. The rapid jet thinning is critical for achieving the small fiber diameters of electrospun materials. Parameters like applied voltage, solution viscosity, and polymer molecular weight significantly influence thinning. As the charged jet undergoes extensive thinning and whipping instabilities in the electric field, solid polymer fibers are formed and collected on the grounded metal collector [42]. The evaporation of solvent from the jet causes the solidification of the polymer solution into continuous fibers. The thinning jet’s high surface area-to-volume ratio promotes rapid solvent evaporation, transitioning the solution into solid fibers within milliseconds. The dried and solidified fibers accumulate on the collecting plate, with the grounded electrode helping to dissipate any residual charge [43]. The accumulation of solid mats on the collector is the final step that enables electrospinning to mass-produce nonwoven polymeric materials for various applications [42,44].

**Figure 1:**
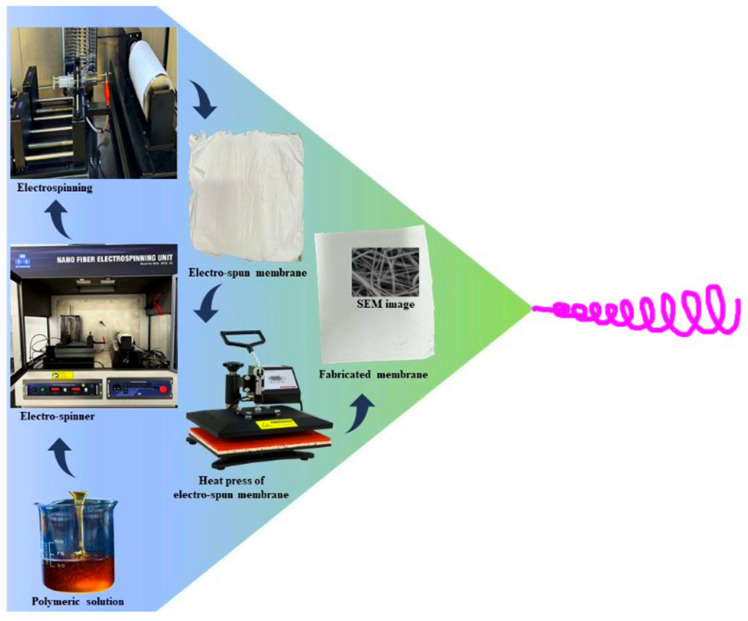
Schematic representation of electrospun membrane fabrication.

The electrospinning setup consists of a syringe containing the polymer solution, a high-voltage power supply of 30 kV, and a grounded collector for fiber deposition. The optimized parameters include a syringe diameter of 10 mm, a polymer volume of 10 mL, a tip-to-collector distance of 75 mm, and a controlled flow rate of 30 μL/min. These conditions ensure uniform fiber formation, improved membrane porosity, and enhanced mechanical stability, contributing to developing high-performance membranes for efficient PW treatment. Figure 1 illustrates the schematic representation of electrospun membrane fabrication.

### 2.3. Membrane Modification

The active layer was created through interfacial polymerization (IP) and requires two solvents. The first consists of 2 g of heptane and 100 mL of TMC. This solution must be created first because it must be cooled to a temperature of −20 °C. Heptane was first weighed and set aside. TMC was weighed, and the vial was stored under nitrogen after use. The heptane and TMC were combined and placed in the freezer at −20 °C. The second solvent was then prepared, consisting of 2 g MPD, 2 g CSA, 1 mL TEA, and 0.2 g SDS. Water was first weighed and set aside. It was then followed by weighing and combining the MPD, CSA, TEA, and SDS, respectively. The membrane was then immersed in the second solvent for ten minutes. Afterward, the membrane is removed, and a rubber roller is used to remove the excess solvent from the membrane. The membrane was then dipped in the first solvent for 30 s for the IP reaction. This membrane was then cured for five minutes at 70 °C. Lastly, it was rinsed in 250 mL of DI water and kept in a DI water bath until further usage.

Active Layer Zwitterionic Coating solutions containing 2.5 g SBMA and 1 g dopamine were prepared with a pH of 8.5 by adding 430 mg of Trizma-HCl salt in 100 mL of pure water. The membrane was fixed to a plate with the active layer up. The solution was poured onto the membrane while on a rocking platform shaker to provide sufficient oxygen for PDA to form on the membrane surface. The membrane was soaked overnight in ultrapure water so that the unbounded PDA and SBMA could detach. The states that deposit time varies depending on the time of the membrane. For MF membranes, one hour is suggested; for UF membranes, 45 min is suggested; and for NF and RO membranes, 30 min is suggested. The support layer zwitterionic coating does not require any pretreatment of the membrane. First, 1.48 g MPC and 2.06 g HS-C_10_-SH were dissolved into 20 mL of degassed chloroform. Then, 27.9 mL di-isopropylamine was added to the solution. The solution was mixed for 20 h at room temperature and then precipitated in acetone. The precipitate is then collected, dried, and dissolved in DI water. The MPC-SH was obtained after freeze-drying. A PDA solution will coat the membrane at room temperature for three hours. This solution will contain 100 mL of Tris buffer solution with a pH of 8.5, which contains 2 mg/mL dopamine-CI (200 mg). The membrane was then coated at room temperature for 12 h with a second solution. This is a 1 mg/mL MPC-SH solution with 1 mL trimethylamine and 100 mL DI water. After the coating is complete, the membrane was immersed in DI water.

### 2.4. Synthetic Produced Water Preparation

The SPW was prepared by first creating individual stock solutions of various chemicals. Specifically, NaCl (200 g), NaHCO_3_ (325 g), Na_2_CO_3_ (20.5 g), Na_2_SO_4_ (1.5 g), CaCl_2_ (6.25 g), and MgCl_2_·6H_2_O (3.95 g) were each separately dissolved in distilled water to make individual 1 L stock solutions. Complete dissolution was ensured through continuous stirring. After labeling and storing these solutions, precise volumes (20 mL each) of the NaCl, NaHCO_3_, Na_2_CO_3_, Na_2_SO_4_, CaCl_2_ and MgCl_2_·6H_2_O stock solutions were combined with approximately 880 mL distilled water, resulting in a 1 L brine solution. For further modification, the prepared brine was mixed with 60 mg Sodium Dodecyl Sulfate (SDS) and heated at 45 °C for one hour, followed by the addition of 5 mL hydra pump oil. This mixture was vigorously stirred at 1200 rpm for 45 min and subsequently sonicated for 30 min using a probe sonicator to ensure homogeneity. Finally, the prepared SPW was stored in 2 L glass bottles for subsequent experimental use [45].

### 2.5. Forward Osmosis

**Figure 2:**
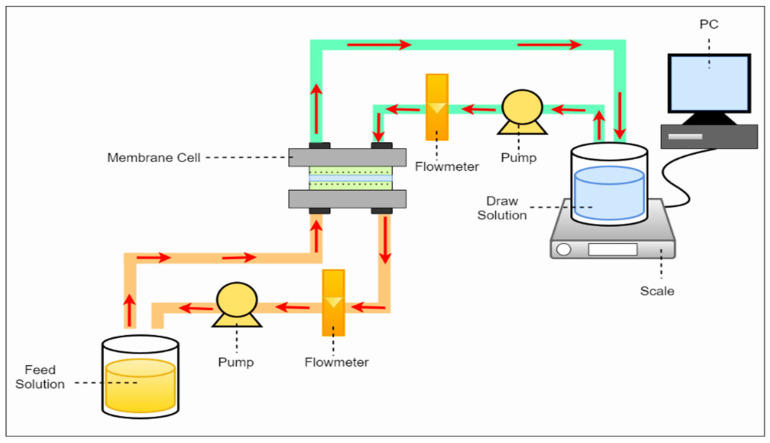
Schematic diagram of the laboratory-scale forward osmosis system.

This study employed a lab-scale forward osmosis system, as illustrated in Figure 2. The system comprises a membrane cell, two circulating gear pumps, feed and draw solution reservoirs, a digital balance, and a computer for real-time data acquisition. When the FO membrane was positioned at the center of the membrane cell, two symmetric flow channels are established on either side, facilitating water transport driven by the osmotic gradient. The FO membrane was operated in the active layer facing the feed solution. The reduction in adequate osmotic pressure is governed by the Van’t Hoff Equation (1) due to internal concentration polarization was quantified using the membrane performance ratio (P*_m_*), defined as the ratio of the experimentally measured water flux (J*_exp_*) to the theoretically predicted flux (J*_th_*_r_), calculated based on the osmotic pressure difference between the bulk feed and draw Solutions (2).

(1) π=iCRT

where i = Van’t Hoff factor, C = Molar concentration of solute (mol/L), R = Universal gas constant (8.314 J mol^−1^ K^−1^), and T = Absolute temperature (K).

(2) $$P_{m}=\frac{J_{exp}}{J_{thr}}$$

The flow rate was fixed at 1 L per minute (LPM) for SPW and 0.75 LPM for PW after initial optimizing conditions. To monitor membrane performance, the digital balance continuously measures the weight change in the draw solution tank, representing the net permeate volume transferred across the membrane. The computer records these measurements at one-minute intervals and was subsequently used to determine the real-time water flux (J_w_). The water flux was calculated based on the weight change in the draw solution over time, providing a direct evaluation of membrane efficiency under various operating Conditions (3). The total solid rejection (%TS) was calculated using Equation (4), where TS*_Initial feed_* is the initial concentration and TS*_Increased_* is the final concentration of the feed solution after the forward osmosis run.

(3) $$J_{W}=\frac{∆Weight}{Water\;density\times Effective\;area\times ∆Time}$$

(4) $$\%\;TS_{Rejection}=\frac{{TS}_{Initial\;Feed}-{TS}_{Increased}}{{TS}_{Initial\;Feed}}\times 100$$

## 3. Results and Discussion

### 3.1. Membrane Characterization

A range of advanced characterization methods was used to thoroughly assess the structural, chemical, mechanical, and surface properties of the produced membranes. Scanning electron microscopy (SEM) combined with Energy-Dispersive X-ray Spectroscopy (EDS) was utilized to examine the membrane structure, fiber consistency, and elemental makeup. Thermogravimetric Analysis (TGA) evaluated the thermal stability of the electrospun membranes. Fourier Transform Infrared Spectroscopy (FTIR) was utilized to verify the chemical changes at each fabrication phase, pinpointing essential functional groups. X-ray Diffraction (XRD) was used for additional analysis of crystalline behavior, which verified the mainly amorphous characteristics of the membranes. Contact angle measurements were performed with a Cam-Plus contact angle meter to evaluate surface hydrophilicity. The mechanical integrity of the membranes was assessed through tensile strength testing. Ultimately, ^1^H and ^13^C Nuclear Magnetic Resonance (NMR) spectroscopy offered molecular-level validation of effective functionalization and crosslinking, with distinctive chemical shifts linked to aromatic structures, zwitterionic groups.

#### 3.1.1. Optimization of rGO Loading (%)

**Figure 3:**
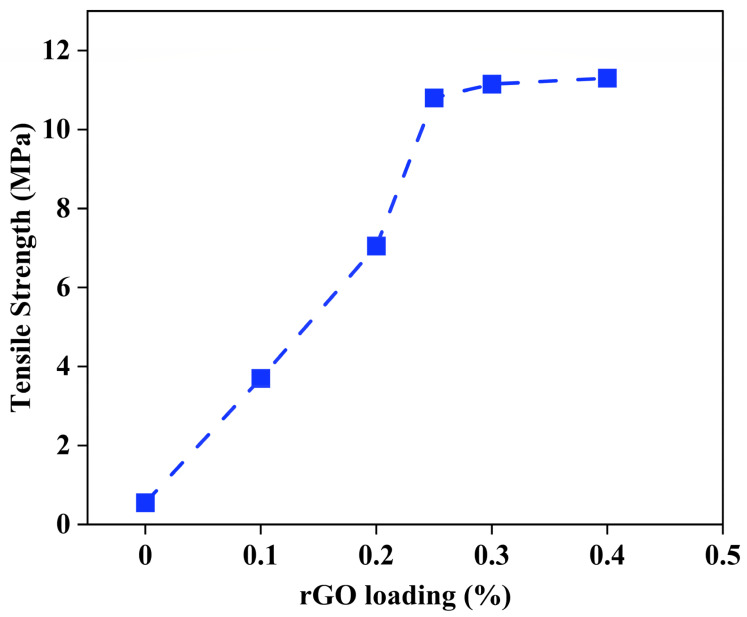
Tensile strength of different rGO loading (%) of the fabricated membrane.

Different amounts starting from 0.10, 0.20, 0.25, 0.30, and 0.40% of rGO were examined to enhance the mechanical strength of the fabricated membrane. Figure 3 shows that the membrane’s tensile strength notably improved with increased rGO content, peaking at about 10.9 MPa for 0.25 wt.%. This improvement is due to the efficient distribution of rGO in the polymer matrix, which aids in load transfer and strengthens the electrospun fibers. Apart from this loading, additional rises in rGO concentration displayed minor enhancement in tensile strength, indicating a saturation threshold where surplus rGO might start to agglomerate or impede consistent fiber formation. Thus, 0.25 wt.% was chosen as the ideal rGO loading for membrane production, compromising mechanical strength and processability while maintaining the electrospinning characteristics and membrane structure. This improved formulation enhances structural strength during FO processes, especially under hydraulic or osmotic stress situations commonly found in PW treatment.

#### 3.1.2. Mechanical Properties

The mechanical characteristics of nanofibrous membranes have been identified as pivotal factors in wastewater treatment. In this study, an examination of the mechanical characteristics as depicted by the tensile curve was conducted for all three nanofiber membranes. The membrane thicknesses were as follows: 127 μm, 154.2 μm, and 228.6 μm, respectively. The variation in thickness observed in the different layers of the membrane can be attributed to several factors inherent in the fabrication process outlined in the experiment. Interfacial polymerization (IP) involves chemical reactions sensitive to reactant concentration, temperature, and reaction time. Variations in these parameters can lead to fluctuations in the thickness of the active layer formed [19].

Additionally, the evaporation of solvents used in the IP process and subsequent coating steps can occur at different rates due to temperature and air exposure. This uneven evaporation may result in non-uniform deposition of materials and, consequently, variations in layer thickness across the membrane surface. Furthermore, the techniques employed for coating, such as dipping or pouring, may introduce variability in the amount of material deposited onto the membrane. This variance in coating technique can influence the overall thickness of the layers. Moreover, the properties of the substrate onto which the layers are deposited play a crucial role. Variations in substrate properties or surface roughness can affect how the layers adhere and spread, leading to differences in thickness. Lastly, the kinetics of chemical reactions during the fabrication process can vary, influencing the extent of polymerization or cross-linking and, subsequently, the thickness of the layers. Considering these factors, the observed decrease and subsequent increase in membrane thickness likely stem from a combination of these elements interacting throughout the fabrication process. The tensile strength confirmed that the raw membrane can withstand 10.9 MPa, the active layer can withstand 12.2 MPa, and the support layer zwitterionic membrane, can withstand 34.4 MPa of force before failing. This pattern indicates that regulated increments in thickness due to interfacial polymerization and zwitterionic surface modification lead to improved mechanical stability. The layered construction and greater material volume enhance stress distribution and improve resistance to mechanical deformation. Moreover, the interactions at the interfaces between layers probably enhance energy dissipation when a load is applied, further strengthening the membranes’ durability.

#### 3.1.3. Contact Angle

The contact angle is the angle at which a liquid/vapor interface meets the solid surface. The contact angle results from the interface/surface tensions (surface free energy) between the liquid and the solid, surrounded by vapor. Contact angle measurement is a quick and easy way to evaluate the cleanness of a solid surface. The main application of contact angle study is to assess the wettability of solid surfaces or the solid/liquid interaction. Other physical properties such as affinity, adhesion, and repellency may also be derived from the contact angle measurements. Cam-Plus Contact Angel Meter- The CAM-Plus is a contact angle meter that provides a patented method (“Tantec’s half-angle”) for quantifying surface energy relative to other measurements on other surfaces. This information makes it possible to predict a surface reaction to coatings accurately.

Tantec’s Half-Angle method is based on the formula for determining contact angles from the droplet dimensions: θ = 2 ∗ arc tan (H/R).

- θ = contact angle
- H= height of droplet
- R = radius of droplet’s base

**Figure 4:**
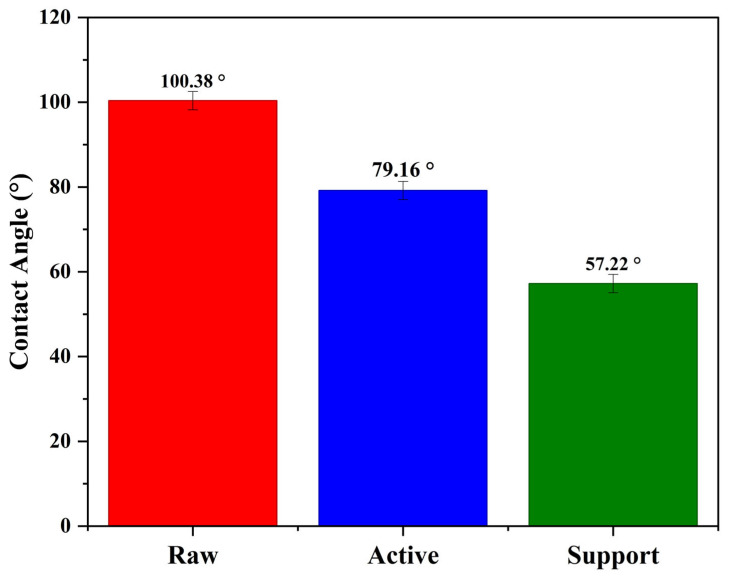
Contact angle (°) of raw, active, and support layer membranes.

Using Tantec’s equation with the dimensions of the droplet on the membrane (θ = 2 ∗ arc tan (6/5)), the contact angle of the membrane is 100.38°. The contact angle was greater than 90°, and the raw membrane had insufficient wettability. Using Tantec’s equation with the dimensions of the droplet on the membrane (θ = 2 ∗ arc tan (3/5.5)), the contact angle of the active membrane was 79.16°, and the contact angle of the support layer zwitterionic membrane is 57.22°. The angle is less than 90°, confirming that both membranes have hydrophilic contact angles (Figure 4).

#### 3.1.4. Scanning Electron Microscopy

**Figure 5:**
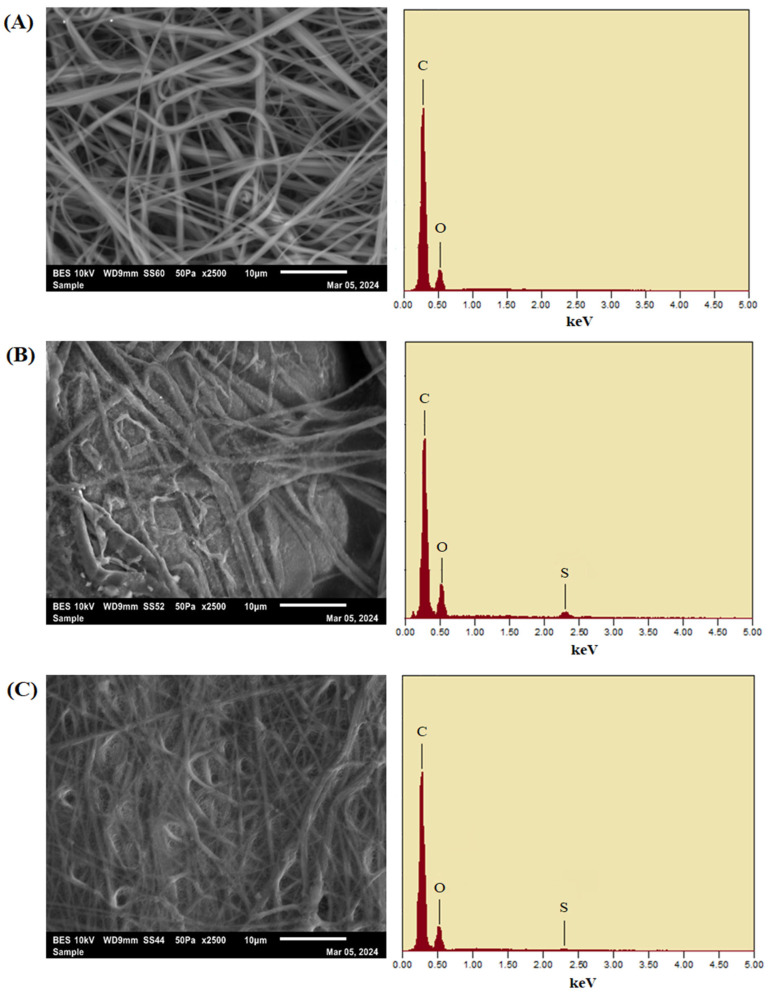
SEM and EDS images of (**A**) raw, (**B**) active, and (**C**) support layer membranes.

The examination of the morphology and fiber diameter of the electrospun membranes using SEM revealed novel and radical findings. The SEM images revealed that the fibers were uniform and smooth, indicating optimal polymer solution preparation and electrospinning parameters with no bead formation (Figure 5A). This morphology was consistent with the initial blend of PEI polymer, DMF/NMP solvent system, and rGO nanofiller. As expected, increasing the polymer concentration increased fiber diameter, a common characteristic of electrospun polymer fibers. This increase can be attributed to several factors contributing to our understanding of the electrospinning process. Firstly, a higher polymer concentration increases the solution’s viscosity, reducing the stretching of fibers in the Taylor cone. Additionally, the ratio of solvent to polymer decreases with more polymer and less solvent. As the solvent evaporates during electrospinning, the polymer was less dissolved due to the reduced solvent presence, resulting in less stretching and thicker fibers. Consequently, higher polymer concentrations lead to thicker fiber formation due to the reduced solvent content per polymer unit. The EDS spectrum obtained verifies that the elemental makeup mainly comprises carbon (C) and oxygen (O), which aligns with the PEI backbone and the oxygenated functional groups of rGO. The lack of extra elemental signals confirmed that no surface functionalization has been implemented at this point.

The SEM images of coated membranes (Figure 5B,C) reveal the presence of a coating on the active layer and support layers on the surface of the nanofibers. After interfacial polymerization, the SEM image of the active layer membrane reveals a more compact fiber network with surfaces that are partly fused and rough. This morphological transformation was due to the creation of the polyamide selective layer resulting from the reaction of MPD with TMC, along with the introduction of the zwitterionic coating made of SBMA and PDA. The thicker surface structure is essential for boosting selective permeability and enhancing antifouling characteristics. EDS analysis reveals significant peaks for carbon and oxygen, akin to the raw membrane, but with a clear presence of sulfur (S) peaks. The occurrence of sulfur was linked to the addition of SBMA, a zwitterionic monomer featuring sulfonate functional groups. The support layer membrane showed a densely intertwined fibrous arrangement with a noticeably rougher surface than the original membrane. The unique surface morphology arises from a two-step coating procedure that includes a PDA pre-coating and subsequent grafting of MPC-SH zwitterionic polymer. The roughness of the membrane’s surface improved hydrophilicity and supports beneficial water transport behaviors. In line with this, the EDS spectrum from the support layer membrane showed notable carbon and oxygen signals, as well as a clear sulfur peak. The existence of sulfur verifies the effective integration of thiol-functionalized zwitterionic coatings from MPC-SH, which are chemically bonded to the PDA-modified surface. The elemental distribution confirms the membrane’s structural soundness and functionalization, designed for antifouling efficacy and mechanical robustness.

#### 3.1.5. Thermogravimetric Analysis

**Figure 6:**
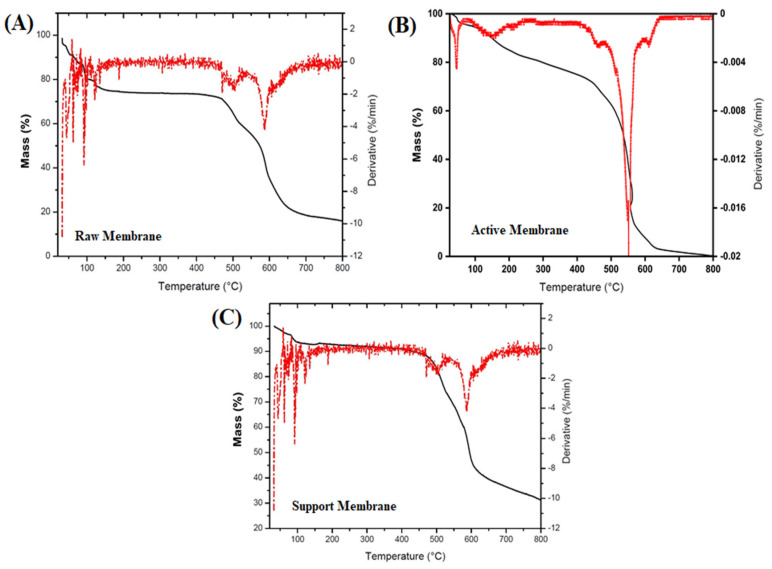
TGA/DTG graphs of (**A**) raw, (**B**) active, and (**C**) support layer membranes.

The thermal stability of the electrospun membranes was assessed through TGA, as shown in Figure 6A–C. The TGA curves for all three membranes raw, active, and support display multistep degradation patterns, offering important information about their thermal decomposition characteristics. For the raw membrane (Figure 6A), three separate mass loss areas were identified. The initial degradation phase occurring between 40 and 120 °C is due to the evaporation of physically adsorbed DMF. The second phase, taking place between 120 and 180 °C, relates to the evaporation of remaining NMP captured in the nanofibrous structure. A significant decomposition event is observed between 470 and 600 °C, linked to the thermal breakdown of the PEI polymer backbone, highlighting its main thermal threshold. The active layer membrane (Figure 6B) displays a later and more pronounced thermal degradation onset beyond 500 °C, which is due to the development of a compact polyamide layer through the interfacial polymerization of MPD and TMC. This layer improves the overall thermal resistance, as shown by diminished mass loss under 200 °C. A steeper degradation slope in the 500–650 °C range indicates heightened thermal rigidity from aromatic cross-linked polyamide structures. Conversely, the support layer membrane (Figure 6C), treated with polydopamine and MPC-SH zwitterionic coatings, shows marginally reduced thermal stability at elevated temperatures. Although the initial weight loss due to the evaporation of residual solvent is comparable, a wider degradation transition between 450 and 600 °C indicates the joint degradation of PEI and zwitterionic layers. The derivative TGA curves additionally validate the unique decomposition kinetics of every layer. The TGA results validate that although all membranes preserve thermal stability up to 400 °C, alterations particularly polyamide crosslinking and zwitterionic coatings greatly affect the thermal degradation characteristics.

#### 3.1.6. Fourier Transform Infrared Spectroscopy

**Figure 7:**
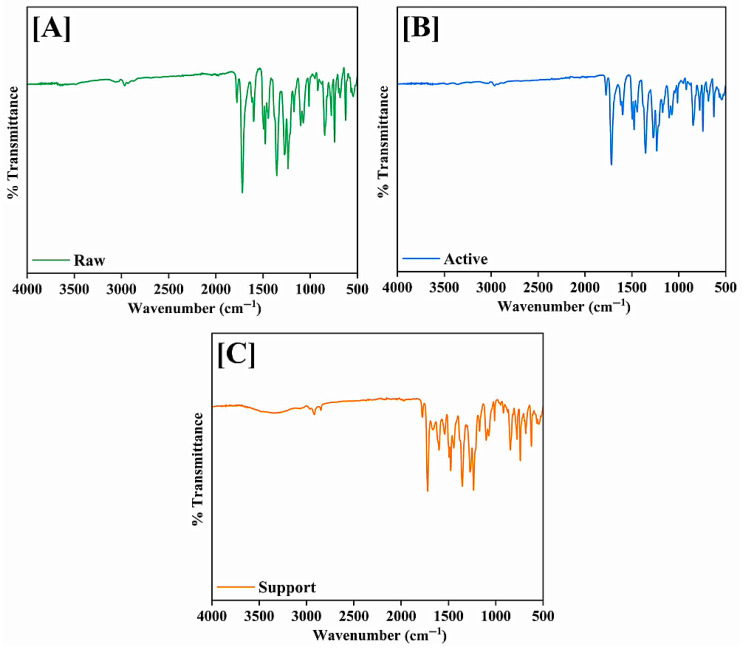
FTIR Spectra of (**A**) raw, (**B**) active, and (**C**) support layer membranes.

The FTIR spectroscopy of the raw, active, and support membranes provided significant insight into the chemical modifications achieved through the sequential fabrication and modification steps (Figure 7A–C). The additional rGO contributed slightly to the distinctive peaks in the raw membrane, which were primarily linked to the fundamental polymeric matrix of PEI. N-H bond stretching vibrations and potential O–H groups from moisture or leftover solvents were responsible for the broad absorption in the ~3300–3400 cm^−1^ region. Peaks at about 1650 cm^−1^ were ascribed to C=O stretching from carbonyl groups, which most likely come from the rGO functionalities or the polymer backbone. The bending vibrations of the CH_2_ groups that were intrinsic to PEI are reflected in the bands about ~1450–1500 cm^−1^. Slight spectrum differences were revealed by the active membrane, which is made via interfacial polymerization (IP) of MPD and TMC and then modified with zwitterionic SBMA-PDA coating. Retained N–H or O–H functionalities were indicated by the broad peak that was still present at about 3300 cm^−1^. The successful synthesis of the polyamide layer during the IP stage was confirmed by the appearance of enhanced absorbance around ~1650–1660 cm^−1^, which corresponded to the newly created amide bonds. S=O stretching vibrations were also responsible for the slight intensity increases around ~1200–1250 cm^−1^ and ~1050–1100 cm^−1^, which validated the grafting of the zwitterionic SBMA groups. These modifications confirm that the surface functionalization intended to improve hydrophilicity and fouling resistance is effective. The support membrane spectrum demonstrated distinct features resulting from the MPC-SH and PDA coating. Similarly to the active membrane, a wide absorption band at about 3300 cm^−1^ was shown, indicating that hydroxyl and amine functionalities are present. More significantly, P–O–C stretching vibrations from the phosphorylcholine moieties added by the MPC-SH grafting were linked to the prominent bands in the area of around 1250–1050 cm^−1^. These features were essential for improving water compatibility and adding antifouling properties. The band near 1550 cm^−1^ was attributed primarily to the N–H bending vibrations (amide II band) and aromatic C=C stretching present in the polydopamine (PDA) coating applied during the support membrane modification. Polydopamine is rich in catechol and amine groups, which contribute to this characteristic absorption. The MPC-SH zwitterionic compound contains ester functional groups (from the methacrylate moiety). A distinct peak in the higher end of this region (around ~1730–1750 cm^−1^) corresponded to ester carbonyl stretching, confirming the successful grafting of MPC-SH onto the membrane. Furthermore, minor peak shifts and intensification in the lower wavenumber range (~700–900 cm^−1^) indicated that the thiol-based anchoring groups (HS-C10-SH), which covalently connect the zwitterionic layers to the substrate, have been successfully integrated. The XRD and NMR spectra further confirm the successful fabrication and modification process.

#### 3.1.7. X-Ray Diffraction

**Figure 8:**
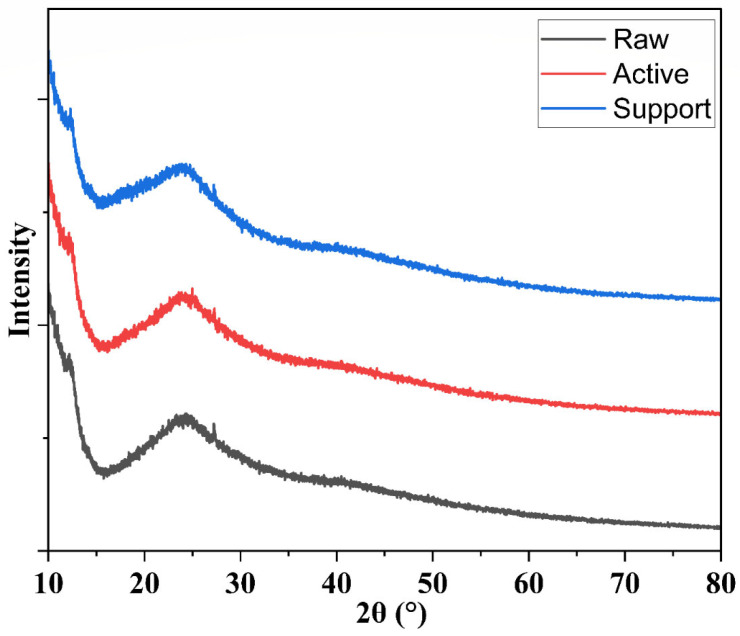
XRD patterns of raw, active and support layer membranes.

The XRD patterns of the three membranes—raw, active layer, and support layer—were evident in Figure 8. The raw membrane showed a broad, low-intensity halo typical of amorphous or semi-amorphous polymeric materials, indicating minimal crystalline domains. By contrast, the active layer membrane (formed via interfacial polymerization) exhibited a slightly more pronounced diffraction pattern in the low-to-mid 2θ region, suggesting the emergence of some ordered domains associated with the newly formed polyamide network. Finally, the zwitterionic support-layer membrane presented additional weak reflections superimposed on the broad polymer background. This may be attributed to partial ordering introduced by the zwitterionic functional groups or any inorganic/ionic interactions in the coating. These changes confirmed the semi-crystalline, predominantly amorphous nature of the electrospun membranes, validating that surface modifications, such as the addition of zwitterionic functional groups, alter the internal polymer arrangement without drastically changing the base crystalline structure. This structural stability, combined with tailored functionalization, optimizes membrane performance in water treatment applications.

#### 3.1.8. Nuclear Magnetic Resonance Spectroscopy

**Figure 9:**
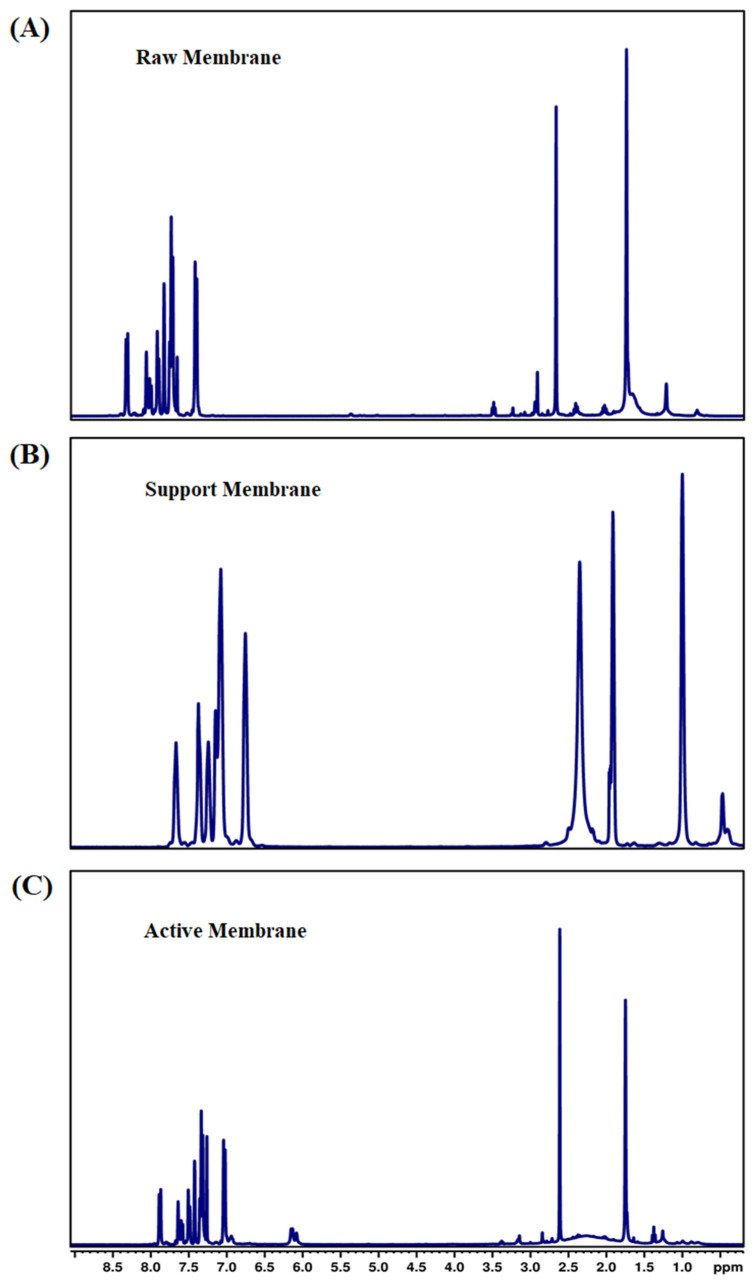
The ^1^H NMR of (**A**) raw, (**B**) support, and (**C**) active layer membranes.

Approximately 15–50 mg of HDO reaction mix was dissolved in 0.5 mL CC_l3_D in a glass NMR test tube. All experiments were carried out on a 400 MHz Bruker Ascend EVO 400 instrument (Bruker Corporation, Billerica, MA, USA). For ^1^H NMR, 32 scans were obtained per sample at a free induction decay (FID) resolution of 0.25 Hz and at 3.99 s acquisition time. For ^13^C NMR, 1024 scans were obtained per sample at a free induction decay (FID) resolution of 0.72 Hz and 1.37 s acquisition time. Topspin v. 3.6.3 software was used for data analysis. The ^1^H NMR spectrum of the electrospun raw membrane revealed characteristic proton signals associated with the polymer backbone and functional groups in the membrane material (Figure 9A–C).

Multiple peaks between 6.5 and 8.0 ppm confirmed an aromatic polymer backbone. Overlapping peaks with the DMF formyl proton (around 7.9–8.0 ppm) can make assigning every aromatic resonance precisely challenging. In many polymer membranes, the aromatic signals were relatively broad due to restricted chain mobility in the solid or semi-solid state, partial insolubility, or strong intermolecular interactions (e.g., hydrogen bonding or dipole–dipole interactions in the polymer matrix). Even though rGO was added to the polymer solution, its ^1^H NMR signature was typically not visible in the final membrane. rGO’s functional groups (e.g., hydroxyls, epoxides) may produce broad, low-intensity signals or be lost in the baseline [46]. The successful formation of aromatic peaks between ~6.5–8.5 ppm from the active layer membrane indicated that the crosslinked aromatic polyamide backbone, broad amide NH region (8–10 ppm), may partially overlap with these aromatic signals. Various small or moderate-intensity peaks can be seen from an aliphatic region (0.5–3.0 ppm) from residual heptane, TEA, SDS, and possibly CSA; the exact pattern depends on the degree to which these additives remain in the membrane vs. being rinsed away. The zwitterionic support layer shows broad peaks for aliphatic chains (0.5–2.0 ppm) from the methylene segments of the polymer backbone and/or the C10 spacer. Broadness increases with polymerization, crosslinking, or strong intermolecular interactions. The most distinctive signal for phosphorylcholine-based zwitterions from quaternary ammonium methyl’s (~3.0–3.3 ppm) and the methacrylate-based backbone (1.6–2.2 ppm) confirmed the zwitterionic polymer. The ^1^H NMR confirmed that a zwitterionic MPC-based coating has been formed on the support. The characteristic quaternary ammonium peak(s), aliphatic chain signals, and any broad peaks from the polydopamine underlayer collectively demonstrated the presence of a dual-layer (PDA + MPC-SH) coating, conferring hydrophilicity and antifouling properties to the membrane support [47,48].

**Figure 10:**
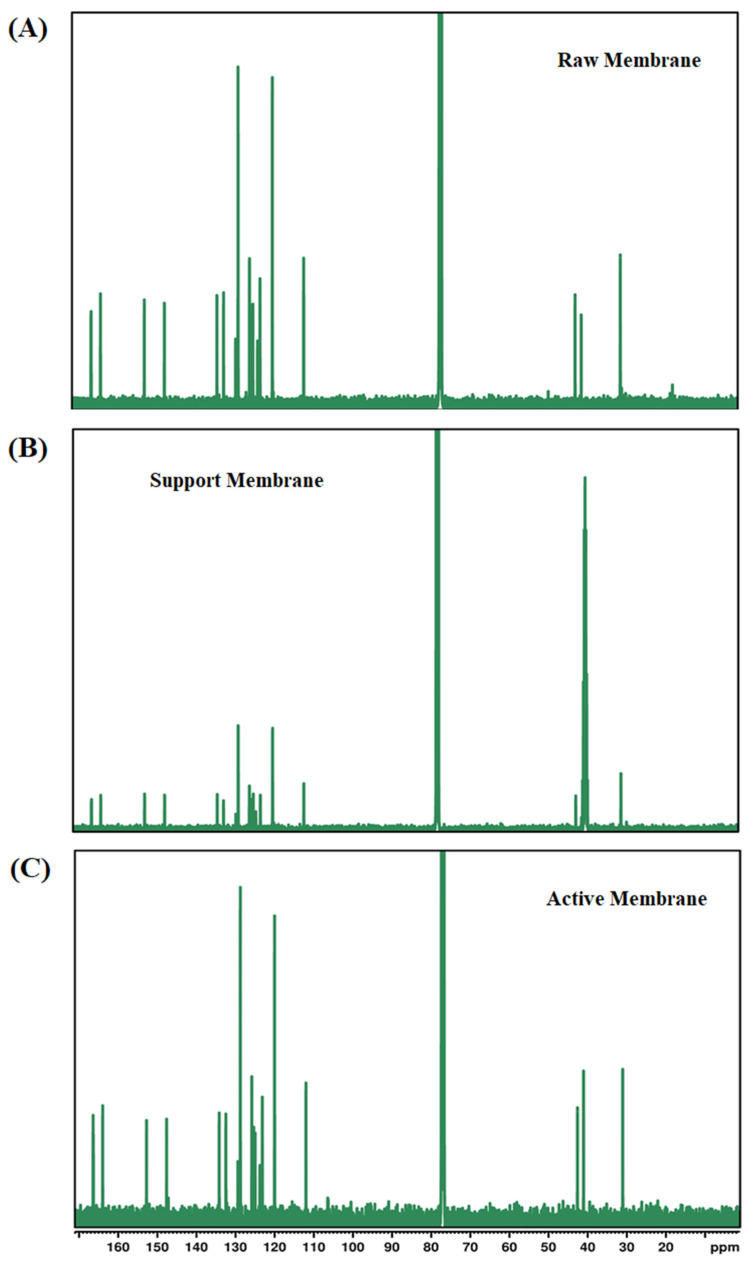
The ^13^C NMR of (**A**) raw, (**B**) support, and (**C**) active layer membranes.

The provided ^13^C NMR spectra reveal structural insights into the raw, support, and active layer membranes (Figure 10A–C). The raw membrane’s spectrum illustrated prominent aromatic peaks at approximately 120–140 ppm, indicative of the polyetherimide (PEI) backbone, consistent with the aromatic polymer structure. Signals around 70–80 ppm suggested the presence of aliphatic and ether groups (–C–O–C– linkages) originating from the polymer structure and the solvents used (NMP and DMF). These peaks confirmed the complete dissolution and homogeneous mixing of the polymer and solvents in the raw polymer solution. The support membrane spectrum showed diminished aromatic peaks, indicative of structural modification due to surface coatings. The spectrum highlighted a signal around 80 ppm, suggesting dominant aliphatic and ether functionalities. These functionalities were expected from the zwitterionic coating (MPC-SH) and polydopamine (PDA) deposition. Aliphatic signals around 20–40 ppm also confirmed successful grafting of HS-C10-SH, which provides hydrophobic, alkyl chain-based functionalities, enhancing membrane stability and reducing fouling tendencies. The peaks demonstrated successful modification without extensive alteration to the original polymer backbone. The active membrane spectrum prominently features aromatic signals between 120 and 140 ppm, characteristic of the polyamide layer formed through interfacial polymerization (IP) of trimesoyl chloride (TMC) and m-phenylenediamine (MPD). Additionally, peaks around 20–40 ppm highlighted aliphatic structures introduced by the surfactant (SDS), triethylamine (TEA), and camphor sulfonic acid (CSA) used during the IP process. Peaks around 70–80 ppm likely corresponded to zwitterionic groups from SBMA and polydopamine-based modifications on the active surface, essential for improving hydrophilicity and anti-fouling properties [49,50].

#### 3.1.9. FTIR Analysis of Post-Operational FO Membranes

**Figure 11:**
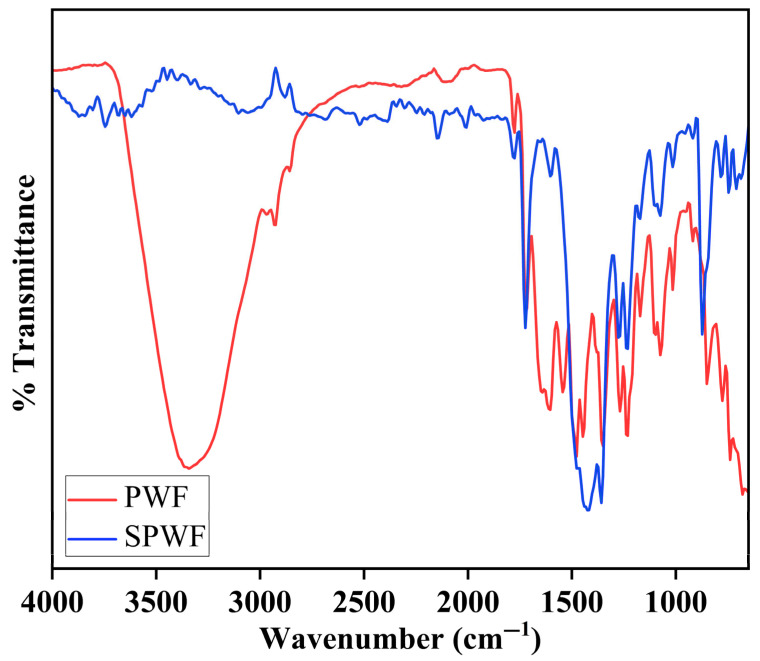
FTIR Spectra of post-operational FO membranes, PWF, and SPWF.

The FTIR spectroscopy was performed to study the surface chemistry of membranes that were fouled after forward osmosis (FO) operation, for the 3 M NaCl draw solute for both SPW and PW (Figure 11). Clear variations in the FTIR spectra indicate the degree and type of foulant accumulation on the membrane surfaces. The membrane contaminated by produced water fouling (PWF) displayed a notably wider and stronger absorption band in the 3300–3400 cm^−1^ range, related to –OH and –NH stretching vibrations. This suggests significant organic fouling, probably from proteins, phenolic substances, and other hydrophilic organics prevalent in real PW. Furthermore, prominent peaks in the 2800–3000 cm^−1^ range were detected, linked to aliphatic C–H stretching vibrations, indicating the buildup of hydrocarbon-based foulants aligned with the existence of residual oil and grease elements in PW. Additional differences were recognized in the fingerprint area. The PWF spectrum exhibited increased absorbance around 1450 cm^−1^ (CH_2_ bending) and in the range of 1030–1100 cm^−1^, which can be linked to C–O and S=O stretching, likely originating from sulfates, sulfonates, and carboxylates introduced by chemical additives or drilling fluids. In comparison, the membrane in contact with Synthetic produced water fouling (SPWF) showed relatively weaker signals in these areas, indicating a reduced level of organic and inorganic fouling, which aligns with the regulated composition of SPW.

### 3.2. Membrane Performance with Forward Osmosis

**Figure 12:**
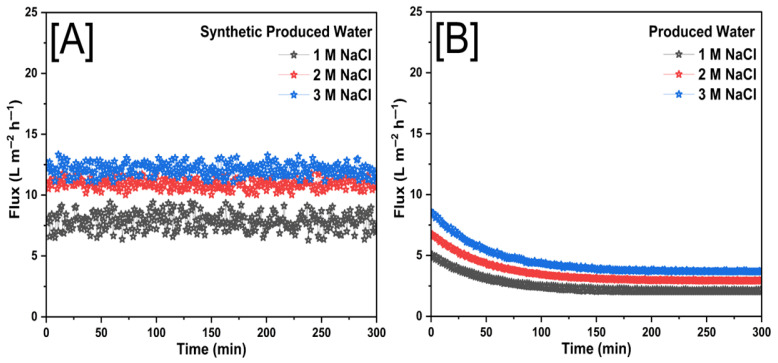
The real time flux of (**A**) SPW and (**B**) PW for the electrospun FO membrane with NaCl as the draw solution at different concentrations of 1 M, 2 M, and 3 M.

**Figure 13:**
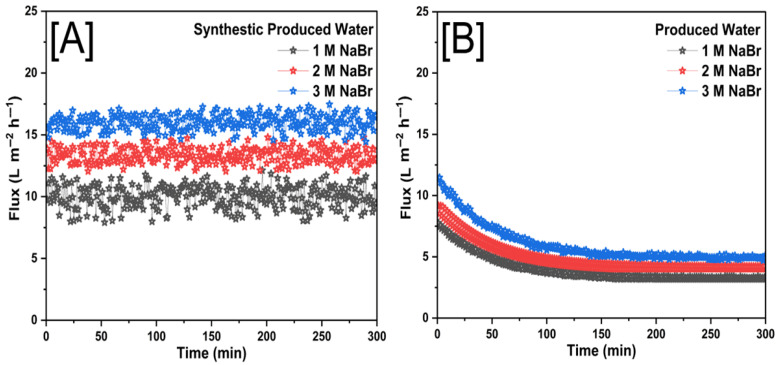
The real time flux of (**A**) SPW and (**B**) PW for the Electrospun FO membrane with NaBr as draw solution at different concentrations of 1 M, 2 M and 3 M.

**Figure 14:**
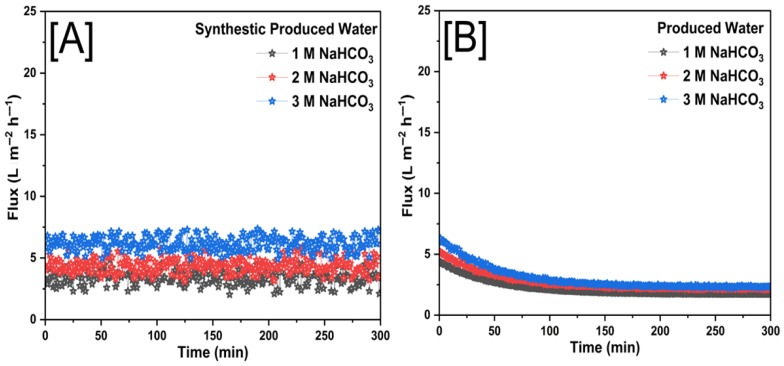
The real time flux of (**A**) SPW and (**B**) PW for the Electrospun FO membrane with NaHCO_3_ as draw solution at different concentrations of 1 M, 2 M and 3 M.

The performance of FO membranes for desalination was evaluated using sodium chloride (NaCl), sodium bromide (NaBr), and sodium bicarbonate (NaHCO_3_) as draw solutes, with a focus on water flux and salt rejection as functions of rGO loading. Figure 12, Figure 13 and Figure 14 illustrate these results. As observed, the water flux increased over time while salt rejection decreased, a trend that aligns with previously reported studies. In FO membranes, a thinner membrane generally results in improved water flux but can compromise structural stability during extended use. To optimize the balance between performance and durability, a 0.25% rGO loading was selected as the ideal concentration for the rGO-based membranes, offering an optimal combination of water flux and salt rejection throughout the study.

The behavior of the permeate water flux was also investigated in this study using PW and SPW as the feed solutions at different concentrations of the draw solution. Concentration polarization (CP) is a phenomenon that causes solutes to accumulate on the active layer of membranes in pressure-driven membrane activities. The osmotic pressure at the membrane surface rises as a result of this solute accumulation, requiring more hydraulic pressure to sustain flux and ultimately decreasing permeate water flux. It should be noted that concentration polarization happens on the feed side of the membrane during FO and is not exclusive to pressure-driven systems. This phenomenon can explain the decreased performance ratios in certain situations.

Throughout the experiments, the flux remained constant for SPW, whereas flux decline appeared linear across the three draw solute concentrations tested for PW. The difference in flux behavior between SPW and PW suggests that real produced water compositions introduce additional challenges, likely due to the presence of complex organic and inorganic foulants, which contribute to more pronounced membrane fouling and scaling. Notably, the zwitterionic modification of the FO membrane surface demonstrated a significantly positive impact on treating both SPW and PW, enhancing water flux and salt rejection capabilities. This highlights the effectiveness of surface modifications in improving FO membrane performance for desalination applications. The ability of zwitterionic coatings to repel foulants is particularly beneficial for PW treatment, where organic fouling and scaling can severely impact membrane longevity and efficiency.

Figure 12 shows the flux behavior of FO membranes when treating SPW and PW with NaCl as the draw solution. The comparison demonstrates how both the water ingredients affect membrane performance. The flow trend for SPW is shown in Figure 12A, where the permeate flux was comparatively constant over the course of the experiment for all tested draw solution concentrations (1 M, 2 M, and 3 M NaCl). According to the observed constant flow, SPW permitted prolonged water transport over the membrane without causing appreciable fouling or osmotic resistance. Minor changes in system circumstances can be the cause of the flux variations, but they do not point to a substantial long-term reduction. The flux trend for real PW, on the other hand, is shown in Figure 12B, where a steady and noticeable drop in flux is noted for all draw solution concentrations. This pattern implied that PW’s organic and inorganic pollutants cause fouling and scaling on the membrane surface, which eventually increased the membrane’s resistance to water transport. An exponential decay pattern characterized the flux decline, with a faster decline during the first period before stabilizing at lower flux values. Although higher concentrations (3 M NaCl) showed an initially larger flow before going through a similar declining pattern, the magnitude of the flux reduction is visible across all NaCl concentrations. The challenges involved in treating real PW are highlighted by the comparison of SPW and PW flow trends. While PW adds complex foulants that gradually impair membrane performance, SPW does not impose severe fouling effects. These results emphasized that in order to maintain flow and improve FO efficiency when treating generated water, membrane surface changes, antifouling techniques, or pretreatment approaches are required.

Figure 13 illustrates the flux behavior in FO experiments when using NaBr as the draw solution for both SPW and PW. The trends observed reveal the influence of water composition and draw solution concentration on membrane performance over time. The flux profile in Figure 13A demonstrates a relatively stable trend for all three tested draw solution concentrations (1 M, 2 M, and 3 M NaBr). The minor fluctuations in flux over time indicate that SPW does not significantly contribute to fouling or osmotic resistance, allowing for consistent water transport across the membrane. Higher NaBr concentrations (3 M) result in greater osmotic pressure, leading to higher initial flux values compared to lower concentrations.

The steady nature of the flux suggested minimal accumulation of solutes at the membrane surface, which is characteristic of a system with limited fouling and concentration polarization effects. A markedly different trend is observed in Figure 13B, where flux continuously declined for all NaBr concentrations when PW was used as the feed solution. The steep initial decline, followed by a gradual stabilization, suggests that membrane fouling and concentration polarization effects are more pronounced in PW. The presence of organic and inorganic contaminants in PW likely contributes to fouling, scaling, and osmotic resistance, which hindered water transport over time. Even at the highest draw solute concentration (3 M NaBr), where an initially greater flux was observed, a significant decline occurs, further reinforcing the impact of PW composition on membrane performance. The comparative results indicated that the composition of PW introduces additional operational challenges in FO systems, leading to more severe flux decline compared to SPW. While NaBr demonstrated strong osmotic efficiency, its effectiveness in maintaining long-term flux stability is influenced by the nature of the feedwater. These findings emphasized the importance of membrane surface modifications, antifouling strategies, and pretreatment methods when treating real PW to sustain flux performance and enhance FO membrane longevity.

Figure 14 illustrates the flux performance of FO membranes when using NaHCO_3_ as the draw solution for both SPW and PW. The flux behavior in Figure 14A shows a relatively stable trend across the experimental duration for all three tested NaHCO_3_ concentrations (1 M, 2 M, and 3 M). The minimal fluctuations suggested that SPW did not contribute significantly to fouling or osmotic resistance, allowing for consistent water transport through the membrane. However, compared to other draw solutes such as NaCl or NaBr, NaHCO_3_ exhibited a lower overall flux, likely due to its lower osmotic pressure at equivalent molar concentrations. The minor variations observed may be attributed to transient system fluctuations but do not indicate a significant decline over time.

In contrast, Figure 14B demonstrates a noticeable flux decline over time for all NaHCO_3_ concentrations when using PW as the feed solution. The initial flux values were higher for the more concentrated draw solutions, but all cases exhibit a gradual decrease, stabilizing at lower flux values as the experiment progresses. This decline can be attributed to organic and inorganic fouling, which accumulated on the membrane surface, increasing resistance to water transport over time. Additionally, NaHCO_3_ was prone to precipitation and scaling, particularly in the presence of divalent cations found in PW, further exacerbating the reduction in membrane performance over time.

The comparative trends revealed that NaHCO_3_, while a viable draw solute, presents certain limitations for FO applications. The relatively lower osmotic pressure of NaHCO_3_ results in lower initial flux values, and its potential for precipitation contributes to scaling and fouling, particularly when treating real PW. Unlike SPW, where flux remained stable, PW introduced additional challenges that accelerate performance decline. These findings underscore the importance of careful draw solute selection, membrane surface modifications, and pretreatment strategies to mitigate fouling and improve FO efficiency when handling complex feedwaters such as PW.

The flux graphs shown represent the performance of FO membranes using NaCl, NaBr, and NaHCO_3_ as draw solutes revealed distinct flux behaviors when treating SPW and PW. In SPW, all three draw solutes exhibited relatively stable flux over time, leading to a constant flux (Jw), while NaBr achieved the highest flux, followed by NaCl, while NaHCO_3_ demonstrated the lowest due to its lower osmotic pressure. However, when treating PW, a pronounced flux decline was observed for all cases, with NaCl and NaBr showing higher initial fluxes but gradually decreasing due to concentration polarization and fouling, which reduces the effective osmotic pressure difference (ΔΠ) over time. NaHCO_3_ exhibited the steepest decline, likely due to its scaling tendencies and lower osmotic efficiency. The results highlight that while NaBr provides superior flux performance, all draw solutes face significant challenges when treating PW, necessitating membrane surface modifications and antifouling strategies to enhance FO system efficiency.

Table 1 summarized the average water flux and percentage of total solids rejection using SPW and PW for three different draw solutions (NaCl, NaBr, and NaHCO_3_) at concentrations of 1 M, 2 M, and 3 M. NaBr consistently showed the highest average flux values for both SPW (9.99–16.05 L m^−2^ h^−1^) and PW (3.96–6.00 L m^−2^ h^−1^) across all concentrations. NaCl displayed moderate flux, with SPW ranging from 7.89 to 12.26 L m^−2^ h^−1^ and PW from 2.54 to 4.46 L m^−2^ h^−1^. NaHCO_3_ presented the lowest flux, recording 2.61–6.22 L m^−2^ h^−1^ for SPW and 2.15–2.97 L m^−2^ h^−1^ for PW. Generally, flux increased with the concentration of the draw solution, regardless of solute type. Total solid rejection decreased with increasing solute concentration across all solutions. NaBr exhibited the highest rejection percentages, reaching 96.02% for SPW and 88.90% for PW at 1 M concentration. NaCl showed moderate rejection percentages, with 92.26% (SPW) and 79.86% (PW) at 1 M, decreasing notably at higher concentrations. NaHCO_3_ had the lowest rejection rates among the tested solutes, with a maximum rejection of 82.12% for SPW and 65.89% for PW at the lowest concentration (1 M). NaBr was the most compelling draw solute in achieving high flux and solid rejection, especially at lower concentrations. NaCl provides a reasonable balance between flux and rejection efficiency. In contrast, NaHCO_3_ demonstrates the least effectiveness as a draw solution under these conditions, showing lower flux and reduced solids rejection performance. Table 2 summarizes different modifications of electrospun FO membranes for desalination.

## 4. Conclusions

In this study, novel forward osmosis membranes featuring electrospun nanofibrous support integrated with innovative dual-layer zwitterionic coatings explicitly designed for PW treatment were successfully fabricated and evaluated. The membranes uniquely combine the structural advantages of electrospun polyethyleneimine (PEI) nanofibers, optimized reduced graphene oxide (rGO) loading, and advanced zwitterionic surface functionalization (SBMA and MPC-SH anchored via PDA chemistry). This innovative dual-layer strategy addresses key limitations of traditional FO membranes, including fouling propensity, low flux, and inadequate rejection performance. Comprehensive membrane characterization confirmed the dual-layer zwitterionic modification on the fabricated membrane surface. The membranes exhibited superior performance in forward osmosis processes, surpassing conventional FO membranes reported in the literature, achieving optimal water flux, high contaminant rejection efficiency, enhanced fouling resistance, and stable long-term operation. Specifically, membranes using NaBr as a draw solution achieved the highest average flux (16.05 L m^−2^ h^−1^ for SPW and 6.00 L m^−2^ h^−1^ for PW) and excellent solid rejection (up to 96.02% for SPW and 88.90% for PW). This research introduces a transformative approach in membrane technology, merging cutting-edge nanofiber fabrication, graphene-based reinforcement, and targeted zwitterionic surface engineering. The results provide critical insights into advanced membrane designs that could significantly influence future research directions and industrial implementation, promoting sustainable water management and environmental protection in challenging wastewater scenarios.

## Figures and Tables

**Table 1:** Average flux and total solid rejection percentage in draw solution of SPW and PW for NaCl, NaBr, and NaHCO_3_.

Draw Solution		Average Flux (L m^−2^ h^−1^)		% Total Solids Rejection	
		SPW	PW	SPW	PW
NaCl	1 M	7.89	2.54	92.26	79.86
	2 M	10.94	3.54	84.79	66.50
	3 M	12.26	4.46	77.80	54.52
NaBr	1 M	9.99	3.96	96.02	88.90
	2 M	13.33	4.89	88.15	74.45
	3 M	16.05	6.00	81.36	59.04
NaHCO_3_	1 M	2.61	2.15	82.12	65.89
	2 M	4.41	2.66	75.89	57.32
	3 M	6.22	2.97	66.36	48.08

**Table 2:** Summary of the modification of electrospun and Salt flux/Salt rejection of FO membranes for desalination.

Electrospun Membrane Materials	Flux (L m^−2^ h^−1^)	Salt Flux/Salt Rejection	Reference
PES	Water = 37.8	97%	[51]
ePET/PSf	Water =1.13	0.23 (g/L)	[52]
PVDF	Water = 30.4	0.21 (g/L)	[53]
PET/PVA	Water = 47.2	9.5 (gMH)	[54]
Nylon 6, 6	Water = 27	0.44 (g/L)	[55]
p-aramid nanofibers	Water = 19.9	98.6%	[56]
PAN/PSf	Water = 38.3	0.27 (g/L)	[57]
PAN	Water = 62.9	0.132 (g/L)	[58]
PVDF-GO	Water = 54.6	0.42 (g/L)	[59]
CA/PVDF	Water = 31.3	0.03 (g/L)	[60]
Zwitterionic TFC	Water = 15	95.7%	[61]
Hollow fiber	Water = 13.0	87.8%	[62]
PAI substrate			
PEI-Zwitterionic	Average water		This study
dual layered	SPW = 16.05	96.02%	
	PW = 6.00	88.90%	

## Data Availability

Data are contained within the article.
